# Response of a continental fault basin to the global OAE1a during the Aptian: Hongmiaozi Basin, Northeast China

**DOI:** 10.1038/s41598-021-86733-x

**Published:** 2021-03-31

**Authors:** Daijun Fan, Xuanlong Shan, Yousif M. Makeen, Wentong He, Siyuan Su, Yibo Wang, Jian Yi, Guoli Hao, Yuting Zhao

**Affiliations:** 1 College of Earth Sciences, Jilin University, Changchun, 130012 China; 2 Shandong Provincial Key Laboratory of Depositional Mineralization and Sedimentary Mineral, Shandong University of Science and Technology, Qingdao, 266590 China; 3 Key Laboratory for Evolution of Past Life and Environment in Northeast Asia (Jilin University), Ministry of Education, Changchun, 130012 Jilin China; 4 Key Laboratory of Urban Geology and Underground Space Resources, Shandong Provincial Bureau of Geology and Mineral Resources, Qingdao, 266000 China; 5 Qingdao Geo-Engineering Surveying Institute (Qingdao Geological Exploration and Development Bureau), Qingdao, 266000 China

**Keywords:** Geochemistry, Geology

## Abstract

This paper presents new research on a lacustrine anoxic event (LAE). These data include stable carbon isotope (δ^13^C_org_), pyrite sulfur isotope (δ^34^S_py_), trace element and biomarker ratios from the Hongmiaozi Basin (North China) and unravel the response of continental lakes under the influence of early Aptian extreme climate conditions. According to the stratigraphic chronology (122–118 Ma) and carbon isotope correlations, terrestrial sediment was influenced by the early Aptian Oceanic Anoxic Event (OAE1a). The results show that the Xiahuapidianzi Group experienced a significant warming process under negative excursions in carbon isotopes due to the influence of increased carbon dioxide partial pressure (pCO_2_). The climate varied from warm and humid to hot and arid (high Sr/Cu, low Rb/Sr, calcareous mudstone), the evaporation and salinity increased (high Sr/Ba and B/Ga), and land input sources decreased (low Zr, Ti and Th). Moreover, high total organic carbon (TOC) content was source from bacteria, algae (n-alkanes), and euxinic depositional environments (Pr/Ph, Cu/Zn and U V Mo). In the stage of continuous carbon isotopes positive excursion, organic matter accumulated rapidly. A paleolake environment model has provided a better understanding of current global climate issues under global warming caused by increased carbon dioxide concentrations.

## Introduction

The distinctive features of the Cretaceous period are global warming, bio-events, shale deposition in marine environments due to intermittent oceanic anoxic events (OAEs) and associated isotopic anomalies^1,2^. The Aptian oceanic anoxic event 1a (OAE1a) at approximately 120 Ma is a typical OAE deposition event. OAE1a is characterized by sedimentary organic matter (Livello Selli) and dramatic fluctuations in the carbon isotope records of carbonate and organic carbon (δ^13^C_carb_ and δ^13^C_org_)^2^. These carbon isotope (δ^13^C) fluctuation records have been interpreted as evidence of perturbations of the global carbon cycle^2–4^. All δ^13^C excursions in δ^13^C_carb_ and δ^13^C_org_ records of OAE1a around the world are mainly preserved in marine basins^5–7^. Given the global significance of the perturbations and the ocean–atmosphere system, their record is found in marine sedimentary matter and terrestrial environments^8^. The terrestrial environment has remained almost unexplored except for the Xiagou Formation^9^ in NW China and Yixian Formation^10^ in NE China. Moreover, the associated research only employed field outcrop section samples and stable carbon isotope method. Drilling core samples with better integrity and continuity are more accurate for analysis of stable isotopes. Therefore, such work cannot fully reflect the detailed changes of the terrestrial sedimentary environment during the anoxic period, which further restricts the use of such data in determining the precise causes and changes mechanisms of the paleo-sedimentary environment under greenhouse.

**Figure 1:**
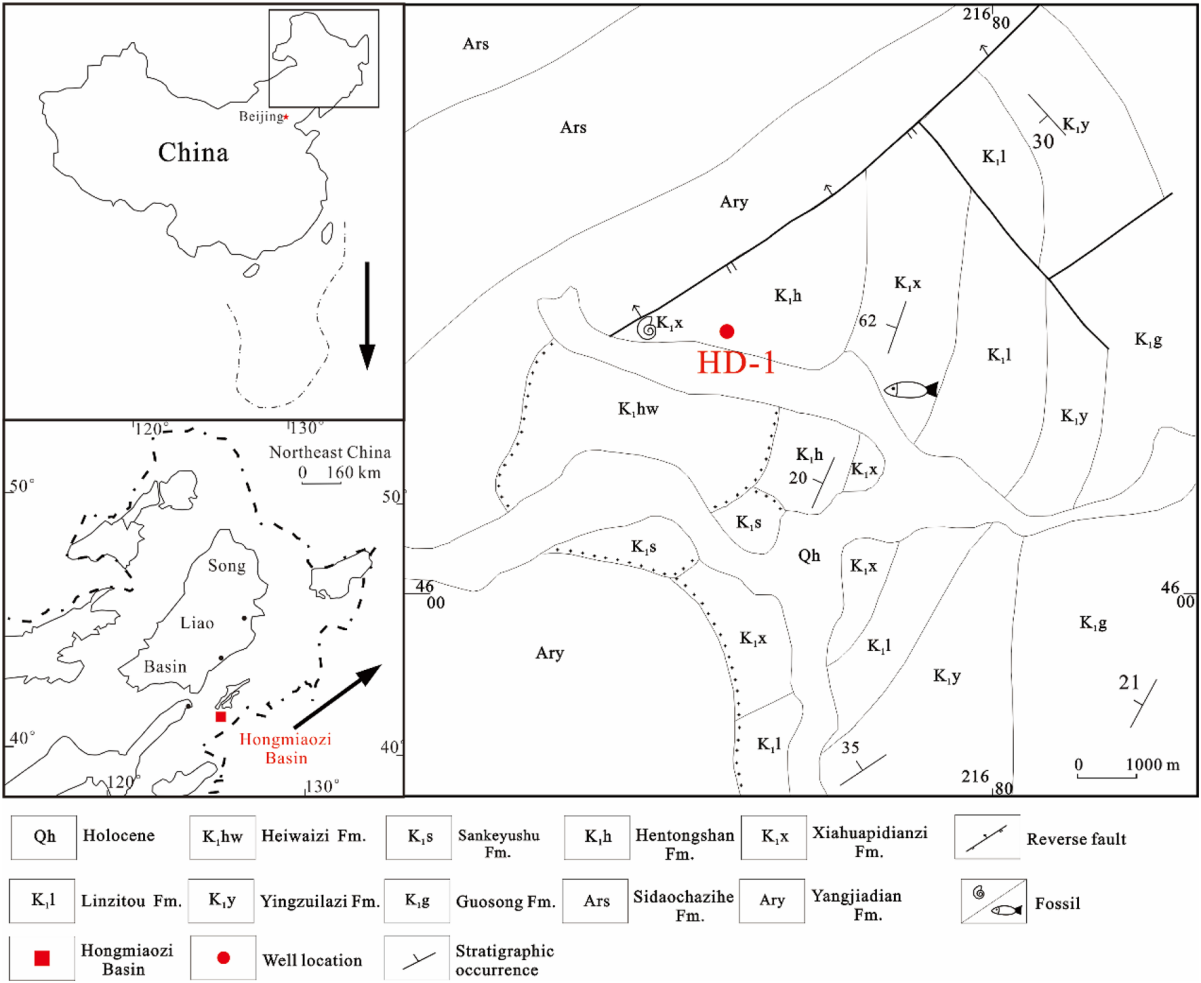
Location of the Hongmiaozi Basin, drilling position and stratum distribution. (This figure was drawn by CorelDRAW Graphics Suite 2019, vision number: 21.3.0.755, url: https://www.corel.com/cn/).

The Xiahuapidianzi group (122–118 Ma) of the Hongmiaozi Basin in the northeastern part of China (Fig. 1) is a typical lacustrine sedimentary stratum. Lake sedimentation is the best carrier to reveal paleoenvironment and paleoclimatic changes because it is very sensitive to climate change^11^. Thus, research on such sedimentation will provide distinct high-resolution records from the Xiahuapidianzi Formation about the paleoenvironment changes within the lacustrine strata.

This study aims to understand the influence of OAE1a on the terrestrial sediment and might also provide a reference for the impact of current lakes on the greenhouse effect through comprehensive indices (e.g., stable isotope geochemistry, element geochemistry, organic geochemistry, etc.). Organic stable carbon isotope and pyrite sulfur isotope methods are widely used for explaining environmental changes (including climate change), bottom-water anoxia and sedimentary environment conditions^1,12,13^, and trace elements are essential in deciphering the depositional environment^14^.

## Geological setting

The Hongmiaozi Basin is located in the Eastern part of the Yilan-Yitong Fault. It covers an area of 290 km^2^ and has a 2.4 km maximum burial depth as determined by the China Geological Survey (CGS). According to the CGS report (in 2015), the basin contains a large number of geophysical and geochemical anomalies for oil and gas. These early achievements act as a foundation for oil and gas exploration and development work.

The geotectonic unit of the Hongmiaozi Basin is a part of the northern margin of the Yingkou-Kuandiantai Arch (III) overlying the JiaoliaoTailong (II) and the Sino Korean paraplatform (I). The tectonic evolution of the Hongmiaozi Basin was mainly influenced by the late Paleozoic Hercynian and Mesozoic Yanshanian movements resulting from the late Middle Jurassic rifting stage (Houjiatun Formation), Early Cretaceous rock melt-filling stage (Changliucun Formation and Guosong Formation), peak volcanic activity stages in the late Early Cretaceous (Yingzuilazi Formation, Xiahuapidianzi Formation and Hengtongshan Formation) and Late Cretaceous rock melt-filling stage (Sankeyushu Formation)^15^.

The target layer (of the studied formation) within the Hongmiaozi Basin is the Lower Cretaceous Xiahuapidianzi Formation. During deposition of this formation, the basin experienced extensional tectonics and a high peak volcanic activity, thus forming a series of fault depressions spreading from the NE and NNE^16^. This tectonic evolution was mainly controlled by the subduction of the Mesozoic and Cenozoic paleo-Pacific plates under the Eurasian plate^17^. In the early stage of the depositional period of the Xiahuapidianzi Formation, the Moho surface was uplifted in the central part of the basin, with significant mantle effect. The studied core samples from Hongdi 1 well reveals that the Xiahuapidianzi Formation contains a set of clastic deposits of lakeshore facies and semi-deep lake facies dominated by dark mudstone and silty mudstone (Fig. 1). In this study, mudstone samples of the Cretaceous Xiahuapidianzi Formation were used to systematically explain the mechanism underlying the effect of anoxic events on the burial lacustrine organic matter in Hongmiaozi Basin by means of GC–MS, trace element analyses, stable carbon analyses and pyrite sulfur isotope geochemistry. This study is important for understanding the influence of OAE1a on the formation of lacustrine black mudstone.

## Materials and methods

The main lithology of the Xiahuapidianzi Formation in the Hongmiaozi Basin consists of mudstone, muddy siltstone, silty mudstone, and sandstone. The location of the Hongdi 1 well is shown in Fig. 1. A total of 35 mudstone core samples were collected to determine the stable carbon (δ^13^C_org_), sulfur (δ^34^S_py_) isotope geochemistry and total organic carbon (TOC) within the studied mudstone. In addition, 7 samples were selected for GC–MS analyses while 24 samples were selected for rare element analyses.

Prior to this analysis, the selected samples were powdered to 200 mesh for Soxhlet extraction analysis to determine the bitumen and hydrocarbon content. The extracted bitumen was separated into asphaltene and maltene fractions. The maltene component separation was carried out using silica gel/alumina column chromatography. Elution with n-hexane, dichloromethane/n-hexane (3:1 by volume) and dichloromethane/methanol (2:1 by volume) was performed to separate saturated hydrocarbons, aromatics and nonhydrocarbon components. Then, the saturated and aromatic hydrocarbons were analyzed by GC–MS on an Agilent 6890GC-5975iMS at the China University of Petroleum (Beijing). Certain specific biomarker peaks, such as n-alkanes, tricyclic terpenoids, hopanes and steroids, were identified based on their retention time and the identification results of previous researchers^18^. The distributions of n-alkanes and isoprene were determined using chromatogram ion m/z 85.

Thirty-two samples were selected for stable carbon isotopic compositions and total organic carbon content (TOC). Firstly, thirty-two samples were crushed to 200 mesh, then weighed respectively. Secondly, the powder samples were acidified with 3 M HCl to completely remove the inorganic carbon (CaCO_3_). Thirdly, washing the samples many times with deionized water to remove the residual HCl traces. Finally, the decarbonated samples were dried in an oven at 80 ℃ for 10 h and reweighed to calculate the percentage of carbonate (TIC) in the bulk samples. About 5–10 mg dried samples were wrapped with folded tin cups, then combustion to CO_2_ with a Euro-vector elemental analyzer (EA) in combination with an Element Isoprime isotope ratio mass spectrometer to determine the TOC abundance and carbon isotopic composition. The carbon isotopic values are expressed on a per mil (‰) basis relative to the Vienna Pee Dee Belemnite standard (V-PDB, δ^13^CV-PDB = 0) within 0.05 ‰ duplicate measurement precision.

The determination method for the sulfur abundance (TS_pyr_) and pyrite sulfur isotope (δ^34^S_py_) composition via Eurovector elemental analyzer in-line with Eurovector Isoprime isotope ratio mass spectrometer (IRMS). The sample of 10 mg was wrapped in a tin cup, and then blown into a quartz tube containing high purity reduced copper by a pure oxygen pulse, heated to 1130 °C. The quartz tube was connected to a Mg (ClO_4_)_2_ desiccant column (remove water), and connected a 3-m stainless steel GC column packed with Porapak-Q heated to 60 °C (separate SO_2_ from other gases). Timed pulses of SO_2_ reference gas (Airgas 99.999% purity, ~ 6 nA) were introduced at the beginning of the run using an injector connected to the IRMS with a fixed open ratio split. The isotope ratio of the reference gas and samples to the determined by ion beam intensities relative to background values. Each five test samples were corrected by two NBS127 and two NZ1 standard samples, and uncertainties for each analytical session based on these standard analyses were better than 1.0% and 0.3‰, respectively, for abundance and isotope compositions. Isotopic results are expressed in the delta notation as per mil (‰) deviations from the Canyon Diablo (V-CDT) standard. This method reference to Cao H et al., 2016^19^. The Stable carbon, pyrite sulfur isotopic and TOC were conducted at the Stable Isotope Laboratory of Oil Shale Test Center of Jilin University.

Trace element were measured at the Oil Shale Test Center of Jilin University by Thermo Scientific ELEMENT high-resolution inductively coupled plasma mass spectrometer (ICP-MS) according to GB/T 14506.30-2010. About 25 mg powder samples were reacted with 1 ml HF and 0.5 ml HNO_3_ in a sealing beaker and dissolution at 185 °C for 24 h. Then, the residues were dissolved with 5 ml HNO_3_ at 130 °C for 3 h. Finally, the remainders were diluted with distilled water to 25 ml for trace element measurement. The result show less than 5% measurement error base on duplicate analysis and standards.

## Result

### Lithology and lithofacies division

**Figure 2:**
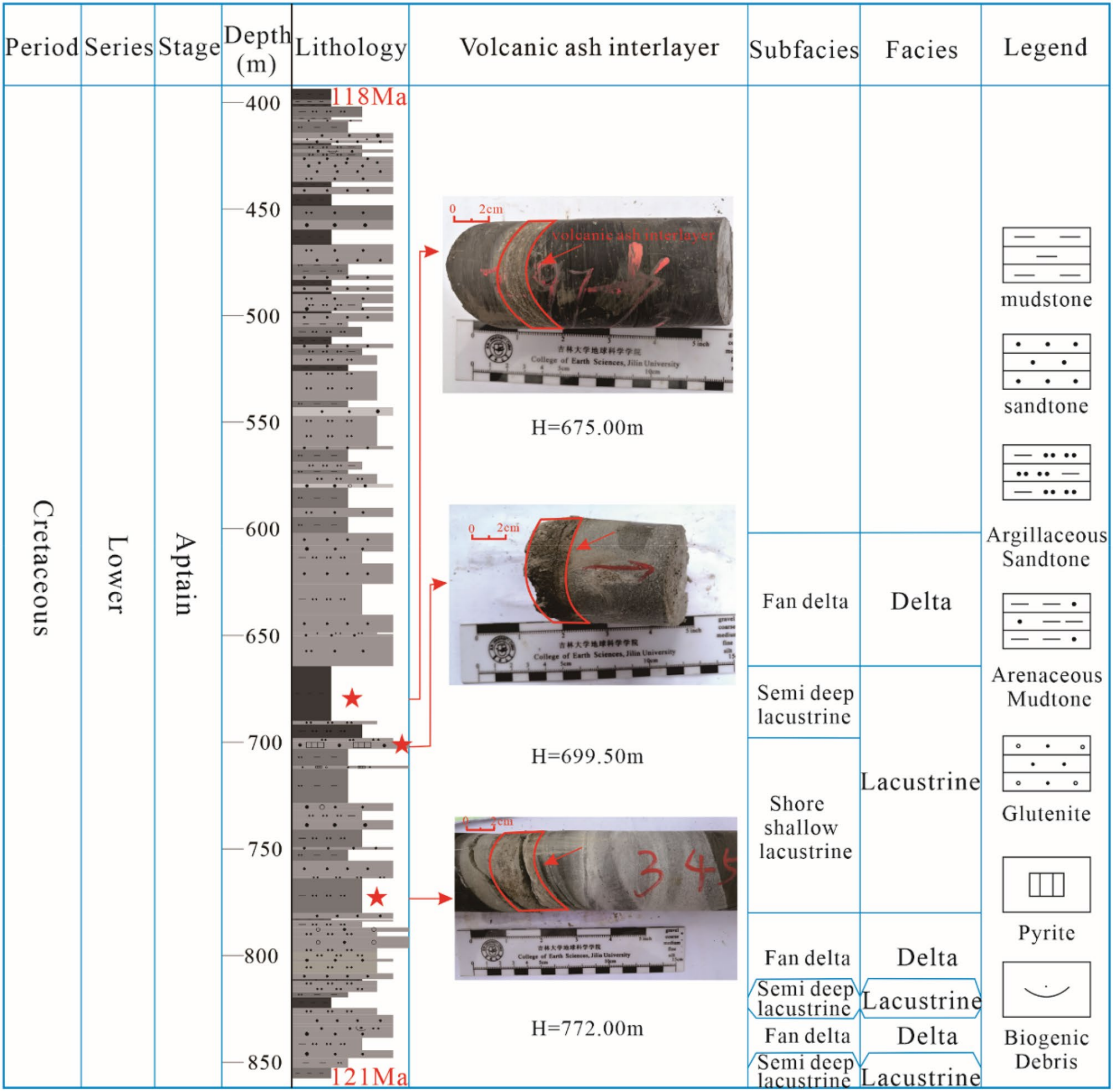
Lithology comprehensive histogram and sedimentary facies division of the Xiahuapidianzi groups. Volcanic ash interlayers in the Xiahuapidianzi Formation sedimentary period (These images were taken by Daijun Fan. This figure was drawn by CorelDRAW Graphics Suite 2019, vision number: 21.3.0.755, url: https://www.corel.com/cn/).

According to the core identification of HD-1 well, the Xiahuapidianzi Formation (Fig. 2) developed black and gray black mudstone, gray silty mudstone, gray silty mudstone, gray siltstone, deep gray argillaceous fine sandstone, light gray and gray fine sandstone, gray and light gray gravel fine sandstone, light gray medium sandstone, light gray gravel medium sandstone, light gray gravel coarse sandstone. According to the lithology combination and variation characteristics, the Xiahuapidianzi Formation was mainly divided into fan-delta front subfacies, shore-shallow lake subfacies and semi-deep lake subfacies.

### Stable carbon isotope and pyrite sulfur isotope

**Figure 3:**
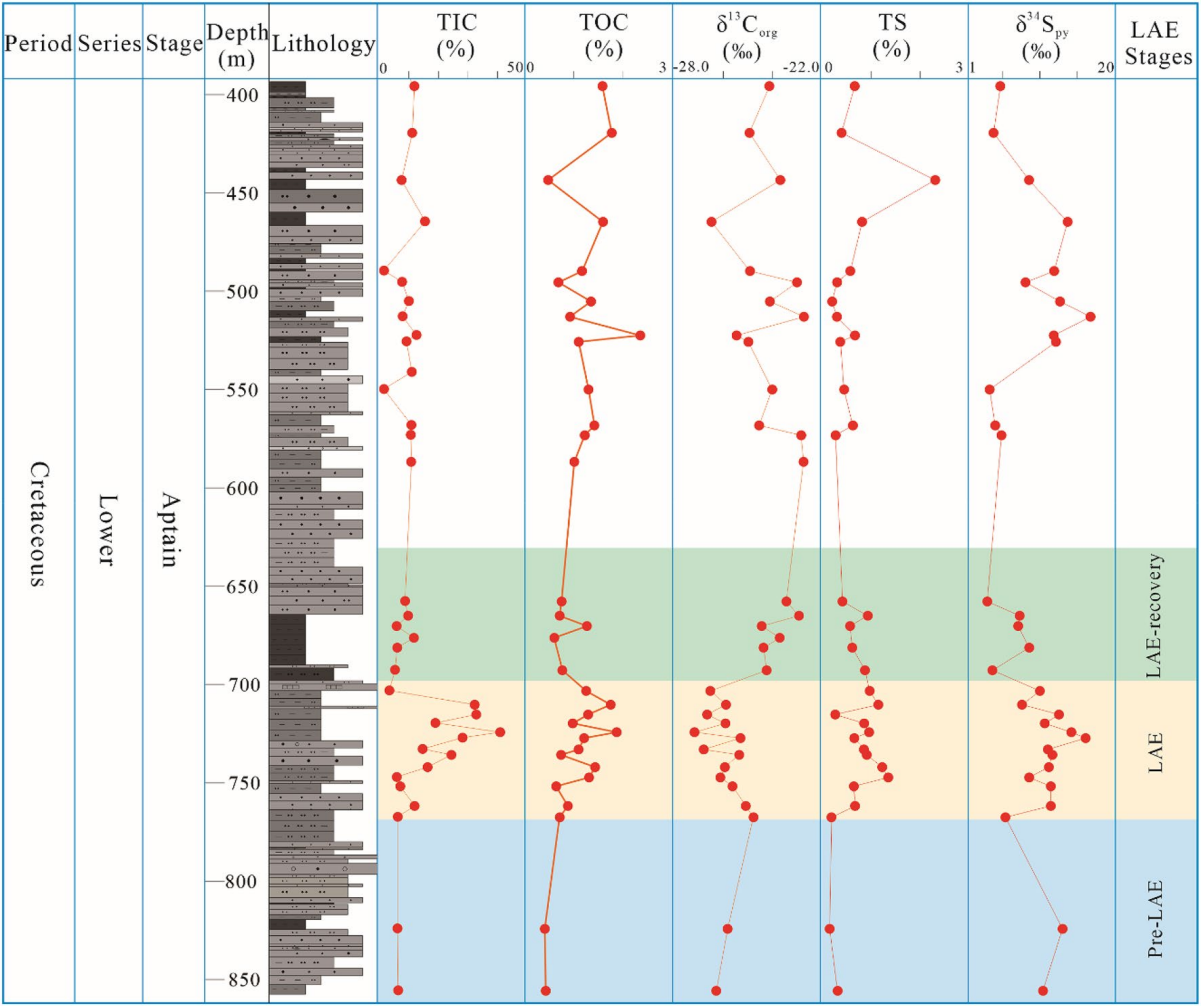
Composite stratigraphic column and vertical variations total inorganic carbon (TIC), total organic carbon (TOC), stable carbon isotope(δ^13^C_org_), total sulfur (TS), and pyrite sulfur isotopes (δ^34^S_py_) of the Xiahuapidianzi Formation mudstones. The stages division of lacustrine anoxia events (LAE) according to obvious stage variation of isotope, lithology and lithofacies distribution and changes, and changes in trace elements. (This figure was drawn by CorelDRAW Graphics Suite 2019, vision number: 21.3.0.755, url: https://www.corel.com/cn/).

The isotopic analysis results of samples from the Xiahuapidianzi Formation are shown in Fig. 3 and Table 1. The organic carbon isotopic (δ^13^C_org)_ values in the study samples are ranging from -27.2% to − 22.7%, with an average of − 24.9%, n = 35. The pyrite sulfur isotope (δ^34^S_py_) values are between 3.7 ‰ and 17.0 ‰, with an average of 10 ‰, n = 33. The TOC values are range from 0.41% to 2.35%, with an average of 1.12%, n = 34. The percentages of TS_pyr_ values are between 0.16% and 2.30%, with an average of 0.69%, n = 33. In Fig. 3, the sampling depth of the δ^13^C_org_, δ^34^S_py_, TOC and TS_pyr_ range from 767.38 m to 724.28 m. The negative values of the δ^13^C_org_ range from − 24.8 ‰ to − 27.2 ‰, showing a 2.4 ‰ decrease, whereas the values of δ^34^S_py_ range from 6.1 ‰ to 14.6 ‰, indicating an 8.5 ‰ increase. The TOC values increase from 0.71% to 1.87%, displaying an increase of about 1.16%. Similarly, the TS_pyr_ values increase from 0.20% to 0.96%, revealing an increase of 0.76%. These values indicate a great change in the sedimentary depositional environment within the Xiahuapidianzi Formation. We could easily distinguish the different stages according to change of the stable carbon isotope, pyrite sulfur isotope, TOC and sedimentary facies (Fig. 3).

**Table 1:** Stable carbon isotope (δ^13^C_org_), total organic carbon (TOC), total inorganic carbonate (TIC), pyrite sulfur isotopes (δ^34^S_py_), and total sulfur (TS) data of the Xiahuapidianzi Formation samples from Hongmiaozi Basin.

Num	Depth(m)	δ^13^C_org_ (‰)	TOC (%)	δ^34^S_pyr_ (‰)	TS (%)	TIC (%)
1	395.80	− 24.1	1.58	5.4	0.67	11.9
2	419.60	− 24.9	1.77	4.5	0.4	11.1
3	443.55	− 23.7	0.48	9.1	2.3	7.5
4	464.65	− 26.5	1.59	14.1	0.82	15.4
5	489.65	− 24.9	1.17	12.3	0.58	1.6
6	495.40	− 23.0	0.69	8.7	0.31	7.7
7	505.15	− 24.1	1.35	13.1	0.21	10.0
8	513.00	− 22.7	0.92	17.0	0.31	7.9
9	522.50	− 25.5	2.35	12.3	0.67	12.6
10	525.65	− 25.0	1.1	12.6	0.38	9.2
11	549.90	− 24.0	1.3	4.0	0.45	11.0
12	568.15	− 24.5	1.42	4.8	0.63	10.8
13	573.20	− 22.8	1.22	5.6	0.28	10.6
14	586.81	− 22.7	1.01	–	–	10.8
15	657.65	− 23.4	0.75	3.7	0.42	8.7
16	665.10	− 22.9	0.71	7.9	0.93	9.7
17	670.30	− 24.4	1.27	7.7	0.57	5.8
18	676.35	− 23.7	–	–	–	11.7
19	681.35	− 24.4	0.6	9.1	0.62	6.1
20	692.72	− 24.2	0.77	4.4	0.88	5.3
21	703.25	− 26.5	1.25	10.5	0.97	3.4
22	710.35	− 25.9	1.75	8.2	1.15	32.2
23	715.28	− 26.7	1.29	13.0	0.27	32.8
24	719.73	− 25.9	0.98	11.1	0.86	19.0
25	724.28	− 27.2	1.87	14.6	0.96	40.9
26	727.15	− 25.3	1.21	16.4	0.66	28.2
27	732.95	− 26.8	1.1	11.5	0.86	14.6
28	735.81	− 25.4	0.74	12.1	0.91	24.4
29	742.08	− 25.9	1.43	11.7	1.23	16.3
30	747.15	− 26.1	1.31	9.1	1.35	5.9
31	751.83	− 25.6	0.64	11.9	0.65	7.1
32	761.80	− 25.1	0.88	11.9	0.68	11.9
33	767.38	− 24.8	0.71	6.1	0.2	6.2
34	824.25	− 25.8	0.41	13.4	0.16	6.1
35	855.65	− 26.3	0.43	10.9	0.32	6.4

The interrelationship of stable carbon isotopes (δ^13^C_org_), pyrite sulfur isotopes (δ^34^S_py_), and TOC can be well correlated by the microbial sulfate reduction (MSR) reaction process as reported by Gill et al*.* and Berner ^13,20^. The MSR can affect most of the organic matter burial and mineralization^21^:

$$ {\text{2CH}}_{{2}} {\text{O}}\, + \,{\text{SO4}}^{{{2} - }} \, \to \,{\text{HS}}^{ - } \, + \,{\text{2HCO}}^{{{3} - }} \, + \,{\text{H}}^{ + } $$

$$ {\text{Fe}}^{{{2} + }} \, + \,{\text{HS}}^{ - } \, \to \,{\text{FeS}}_{{2}} $$

The reaction equations show that sulfate, organic matter, dissolved iron and depositional environment are the decisive factors in the process.

### GC–MS analysis results

**Figure 4:**
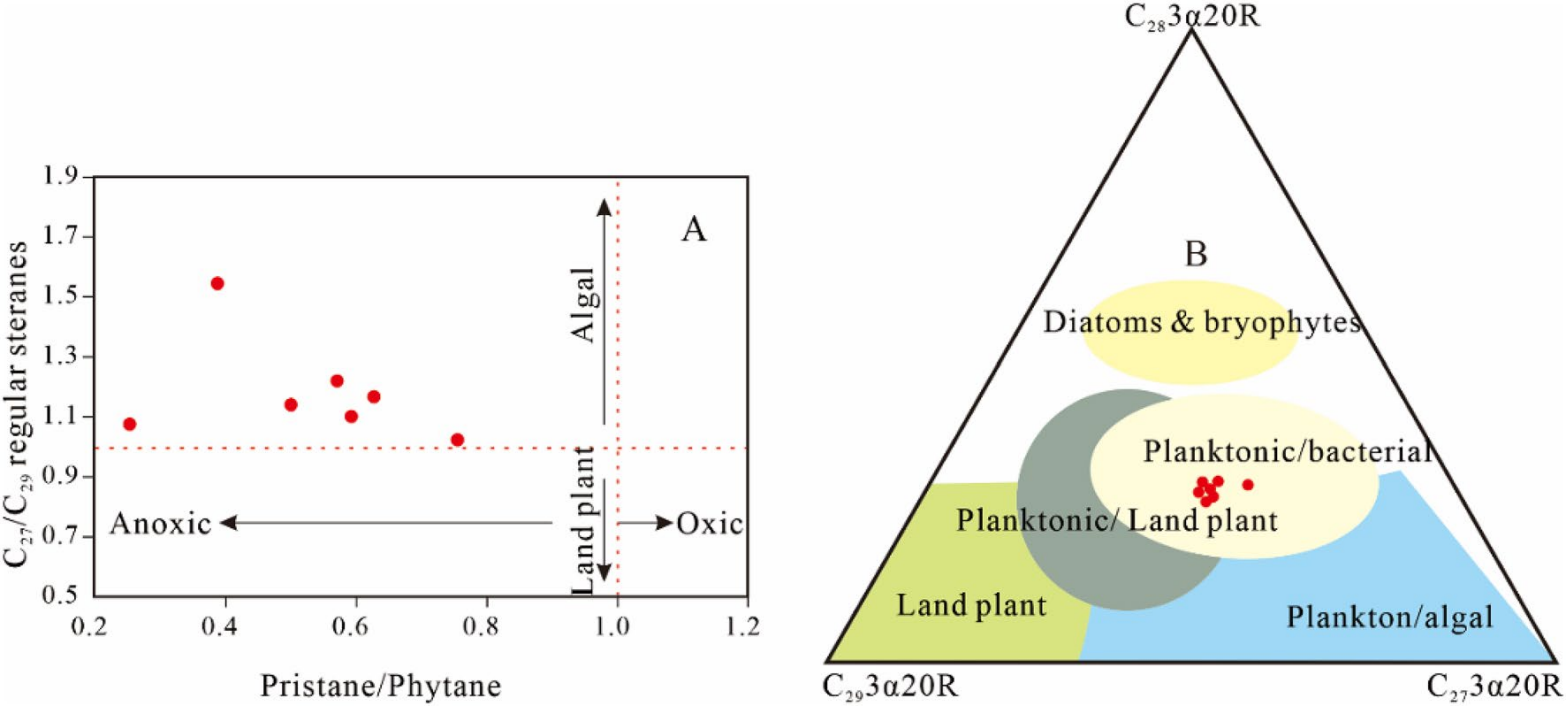
A: Pristane/Phytane versus C27/C29 regular steranes plot. B:C_27_, C_28_, C_29_ααα-20R regular sterane triangular diagram indicating the compositions, organic matter input, and depositional environment for the analyzed Xiahuapidianzi Formation extracts (modified after Makeen, Y. F. et al*.*^28^).

Representative GC–MS signals of the aliphatic fraction with m/z 85 and m/z 191 and m/z 217 ion chromatograms are shown in Fig. 4. The biomarker distribution could well reflect the source input and depositional environment condition of sediments^18^.

**Figure 5:**
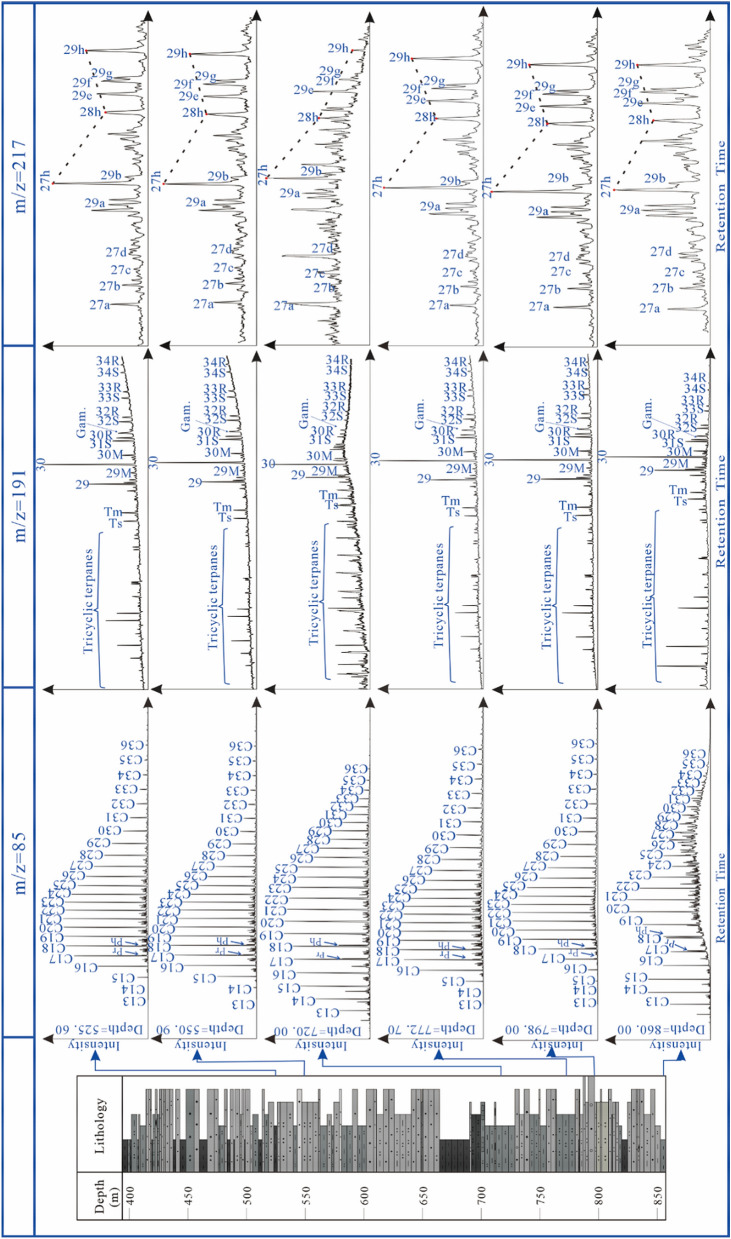
m/z = 85, m/z = 191, and m/z = 217 mass fragmentograms of the saturated hydrocarbon fractions of six representative Xiahuapidianzi Formation mudstone samples.

The *n-*alkanes distribution of the Xiahuapidianzi Formation is illustrated in Fig. 5. The higher molecular weight compounds range from C17 to C23. Tissot and Welte reported that low to medium molecular weight compounds usually received a substantial input of long chain alkanes, either from algae of plant curricular waxes^22^.

The concentration of pristine (Pr) and phytane (Ph) in acyclic isoprenoids are the most important terms^23^. The ratio of pristane to phytane (Pr/Ph) is an indicator of the redox conditions for paleoenvironment conditions^18^. It is generally believed that higher Pr/Ph ratios (> 1) reveal oxidation conditions and low Pr/Ph ratios (< 1) indicate a reduction environment^24^. The Pr/Ph ratio of the Xiahuapidianzi Formation are in the range of 0.25 to 1.01, with an average of 0. 63. The Pr/C17 and Ph/C18 ratios are used to indicate the degree of activity of microorganisms. Generally, the smaller the ratio are, the stronger the activity of microorganisms. The ratio of Pr/C17 and Ph/C18 are 0.04–0.93 and 0.06–1.19, respectively.

The m/z 191 mass fragmentogram of the saturated hydrocarbon fractions of all the analyzed samples displayed high proportions of hopanes relative to tricyclic terpane as shown in Fig. 5. The relatively high tricyclic terpane concentrations in the sediment (Fig. 5 m/z = 191) are consistent with the derivation from lacustrine algal organic matter^25^.

Gammacerane was originally reported as an environmental salinity index for both marine and lacustrine environemts^18^, and the Gammacerane Index (gammacerane/C30 hopane) is believed to reflect the extent of the salinity stratified water column during deposition^26^. The gammacerane/C_30_ hopane ratios range from 0.16 to 0.27, with an average value of 0.22, in the Xiahuapidianzi Formation samples (Table 2).

**Table 2:** n-Alkane and isoprenoids biomarker ratios calculated from TIC, m/z = 191, m/z = 217 mass fragmentogram of selected Xihuapidianzi Formation extracts.

Depth (m)	Pr/C_17_	Ph/C_18_	Pr/Ph	GI/C_30_H	C_27_/C_29_	C_27_ (%)	C_28_ (%)	C_29_ (%)
525.60	0.04	0.06	0.57	0.27	1.22	39.27	28.57	32.16
550.90	0.05	0.06	0.63	0.23	1.17	39.83	26.08	34.09
720.00	0.39	0.36	0.39	0.22	1.54	43.74	27.94	28.32
773.50	0.05	0.08	0.50	0.22	1.14	38.76	27.27	33.97
798.00	0.12	0.23	0.25	0.23	1.08	37.11	28.43	34.47
860.00	0.93	1.19	0.59	0.16	1.10	39.22	25.20	35.58

*Pr* pristine, *Ph* phystane, *GI/C*_*30*_*H* gammacerane index/C_30_hopane, *C*_*27*_*, C*_*28*_*, C*_*29*_ C_27_, C_28_, C_29_ ααα regularsteranes (20R).

The distributions of diasteranes and steranes (C_27_, C_28_ and C_29_) are shown in the m/z 217 ion chromatograms (Fig. 5). The relative abundances of C_27_, C_28_ and C_29_ regular steranes are calculated and the results are given in Table 2. Waples and Machihara documented that the relative distribution of C_27_, C_28_, and C_29_ regular steranes can be used to indicate the source of organic matter input^27^. From Fig. 5, C_27_ sterane presents a dominant abundance in the analyzed samples of the Xiahuapidianzi Formation. The analyzed samples (Table 2) show a high proportion of C_27_ (37.11–43.74%) and C_28_ (25.20–28.57%) compared to C_29_ (28.32–35.58%) steranes. The samples also have relatively high C27/C29 regular sterane ratios in the range of 1.08–1.54 (Table 2). Figure 4A shows that the Xiahuapidianzi Group was sedimentary in a suboxic-anoxic environment and algae were the main source of organic matter. Figure 4B shows the triangular plot of the three end members of the regular steranes and suggests the predominance of planktonic, algal and bacterial organic matter source input to the sediment of the Xiahuapidianzi Formation.

### Trace elements

**Figure 6:**
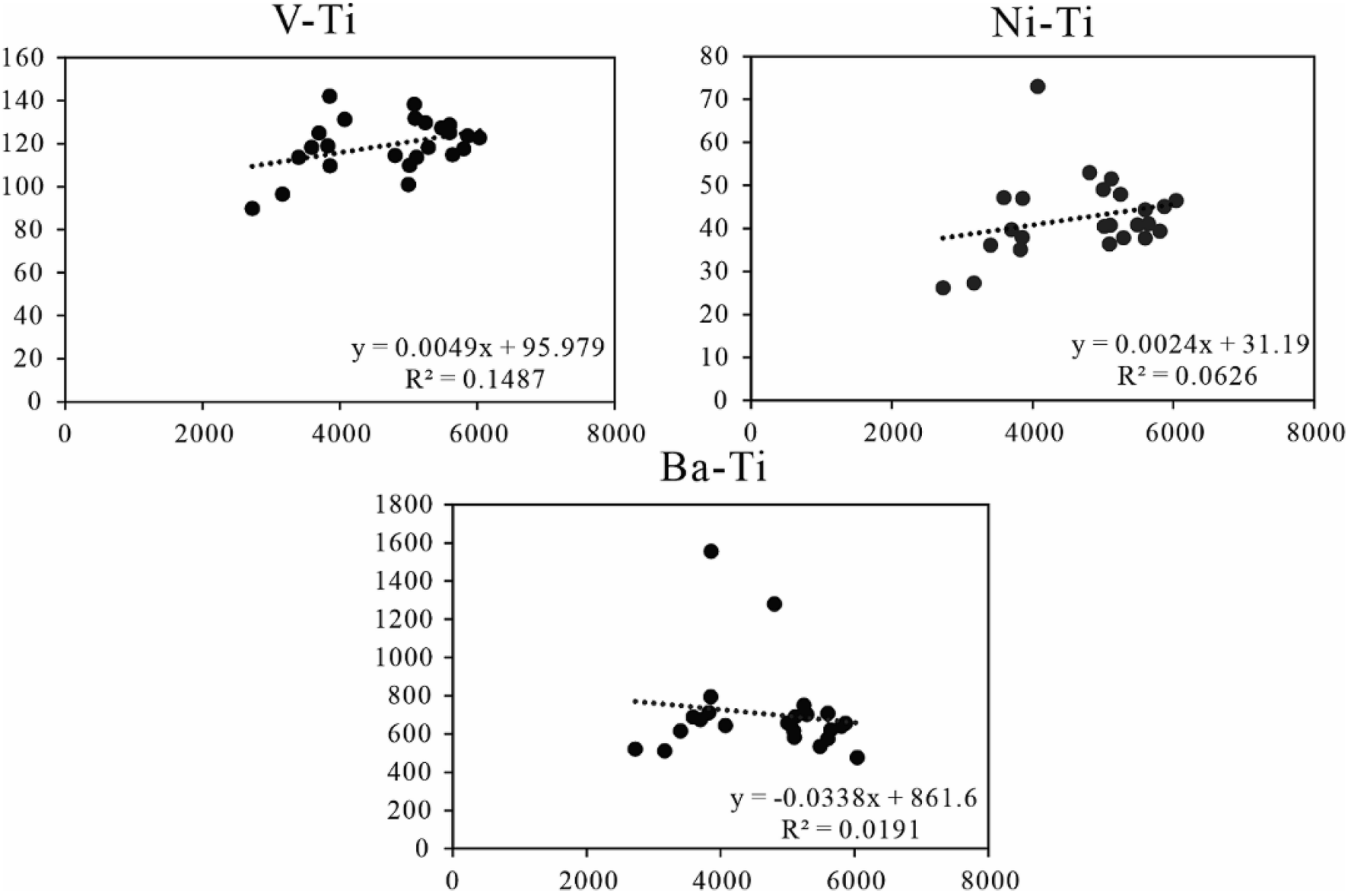
The crossplot of V, Ni, Ba, versus Ti to evaluate the impact of terrigenous debris influence.

Trace element data of the Xiahuapidianzi Formation samples and relevant ratios (e.g., V/(V + Ni), Cu/Zn, and Sr/Ba) are list in Table 3. These elements (e.g., V, Sr, Ba, B, Rb, and Ni) and their ratios are always useful in various studies to decipher the paleoclimate, paleosalinity, and redox conditions during the deposition of sedimentary rocks in basins^14,29–33^. Terrigenous debris are common components in sediments or sedimentary rocks. The impact of terrigenous detrital must be evaluated before the sedimentary environment is judged. The best way to check whether the content of a given element is dominantly controlled by the detrital flux is to crossplot the trace element versus aluminum or titanium, which are commonly overwhelmingly of detrital origin. Here we choose Ti (titanium) for evaluation whether the influence by the detrital flux. Because Ti is commonly overwhelmingly of detrital origin and which are usually immobile during diagenesis^34^. From the crossplot results (Fig. 6), V, Ni, Ba with little R^2^, reflect almost unaffected by terrigenous detrital flux.

**Table 3:** Trace element geochemistry (in μg/g) of the Xiahuapidianzi Formation mudstone samples.

Depth (m)	B	Ti	V	Ni	Cu	Zn	Ga	Rb	Sr	Zr	Ba	Th	U	Mo	Cu/Zn	Sr/Ba	B /Ga	Rb /Sr	Sr /Cu
395.65	70	3404	114	36.1	33.5	92	19.9	157	457	173.2	615	10.7	3.7	3.23	0.37	0.74	3.51	0.34	13.64
464.50	72	3701	125	39.6	37.2	95	21..0	167	481	180.5	675	11.7	3.8	3.47	0.39	0.71	3.44	0.35	12.92
495.25	87	5293	118	37.8	38.0	145	31.0	292	266	294.4	702	16.5	2.7	1.07	0.26	0.38	2.82	1.10	7.01
505.00	85	5020	110	40.5	37.1	135	29.0	254	276	303.6	659	15.1	2.4	1.06	0.28	0.42	2.93	0.92	7.44
512.85	78	5005	101	49.0	33.7	112	26.4	221	318	255.1	658	13.4	2.0	1.02	0.30	0.48	2.96	0.69	9.42
522.35	79	4809	115	52.9	42.5	149	27.6	253	258	236.4	1280	14.2	2.1	6.07	0.29	0.20	2.84	0.98	6.07
525.50	87	5120	114	51.5	37.5	118	27.2	248	348	245.2	691	13.7	2.0	1.46	0.32	0.50	3.20	0.71	9.28
549.75	100	5101	132	40.7	41.6	148	28.6	236	233	245.7	583	13.5	2.3	1.11	0.28	0.40	3.49	1.02	5.59
573.05	100	5870	124	45.1	41.6	127	29.9	289	291	238.9	657	15.4	2.1	1.00	0.33	0.44	3.35	0.99	7.01
586.66	104	5645	115	41.0	31.1	137	28.1	250	244	219.7	622	12.9	2.2	1.07	0.23	0.39	3.69	1.02	7.86
657.50	119	5807	118	39.3	35.7	129	30.1	222	278	260.6	640	14.5	2.2	0.72	0.28	0.43	3.94	0.80	7.77
664.95	143	5602	125	37.6	34.4	135	29.5	212	273	243.3	707	13.5	2.0	0.95	0.26	0.39	4.84	0.78	7.93
710.20	145	3167	96	27.2	33.5	85	16.9	123	685	124.8	511	6.8	2.1	2.30	0.40	1.34	8.58	0.18	20.46
719.58	108	4076	131	73.0	42.5	99	22.0	1640	585	173.0	643	8.4	2.7	7.01	0.43	0.91	4.89	0.28	13.76
724.13	79	2729	90	26.2	29.9	74	14.4	83	894	114.0	522	5.5	2.0	8.18	0.40	1.72	5.48	0.09	29.96
727.00	165	3828	119	35.0	35.6	95	21.1	157	683	144.0	712	8.5	2.5	6.32	0.38	0.96	7.83	0.23	19.17
732.80	90	3859	110	47.0	43.0	83	19.1	129	697	185.2	1555	8.1	3.4	20.66	0.52	0.45	4.71	0.18	16.22
735.66	179	3592	118	47.1	31.4	93	20.7	163	759	141.0	688	10.1	2.6	2.89	0.34	1.10	8.66	0.22	24.18
741.93	142	3856	142	37.8	48.3	99	20.0	151	639	164.6	794	10.4	4.7	3.90	0.49	0.80	7.10	0.24	13.22
767.23	112	5091	138	36.3	48.5	129	32.0	315	316	274.0	617	16.2	2.2	0.58	0.38	0.51	3.51	1.00	6.53
855.50	67	5489	127	40.8	37.0	176	32.9	211	205	362.9	535	15.5	2.6	18.44	0.21	0.38	2.05	1.03	5.54
857.55	74	5602	129	44.3	40.7	186	36.1	224	228	398.9	575	16.7	2.7	20.27	0.22	0.40	2.05	0.98	5.60
858.00	73	6041	123	46.4	36.5	187	36.9	159	197	449.4	477	15.6	3.3	3.64	0.20	0.41	1.99	0.81	5.39
859.83	115	5248	130	47.8	39.1	164	30.1	293	215	259.5	750	14.9	2.0	2.99	0.24	0.29	3.82	1.36	5.51

**Figure 7:**
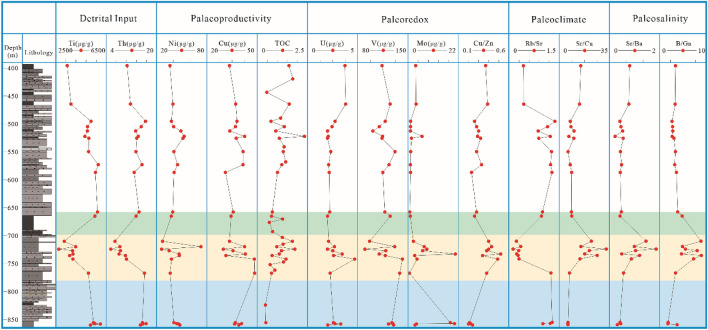
Vertical variations in detrital input indices (Ti, Th), paleoredox indices (U, V, Mo, and Cu/Zn), paleoproductivity indices (Ni, Cu and TOC), paleoclimate indices (Sr/Cu and Rb/Sr), paleosalinity indices (Sr/Ba and B/Ga) for the Xiahuapidianzi Formation in Hongmiaozi basin.

Paleoclimate. Climate is a major factor controlling sedimentary organic matter (OM) input^35^. The climatic conditions can influence the geochemical characteristics of sediments in lacustrine environments by controlling the terrigenous material flux and diagenetic processes^32^. Trace elements Sr, Cu, and Rb are considered to be sensitive to paleoclimate, and Sr/Cu and Rb/Sr ratios may provide useful clues to infer paleoclimatic changes^31^. In dry and hot climates, the lake water area shrinks. In response, Sr becomes enriched in the lake water, while Cu precipitates out. Thus, the Sr/Cu ratio is used to reflect the climate conditions during deposition^36^. Previous studies evaluating palaeoclimate observed consistent results on the ratio of Sr/Cu. When the ratio of Sr/Cu in the range of 1to10, it indicates the climate is humid to semi-humid. When the ratio of Sr/Cu range from 10 to 20, it reveals the climate is semi-humid to semi-arid. When the ratio of Sr/Cu larger than 20, it manifests the climate is arid and hot^37^. The Sr/Cu ratios of this study vary greatly from 5.39 to 29.96, with an average of 11.14, n = 24. The Rb/Sr ratios vary from 0.09 to 1.36, with an average of 0.68, n = 24. The ratios of Sr/Cu and Rb/Sr are generally < 10 and > 0.6, respectively (Table 3), although an obvious change occurs from 710.20 to 741.93 m and 595.65–464.50 m, with Sr/Cu (> 10) and Rb/Sr (< 0.60) (Fig. 7).

Paleosalinity. Trace elements Sr, Ba, Ga, and B are also sensitive to salinity. Sr/Ba and B/Ga ratio can be useful indicators for assessing paleosalinity^29,30,33^. Sr/Ba values < 0.6 indicates a freshwater setting, between 1.0 and 0.6 represent brackish water, and > 1 indicates saline water in an arid climate^38^. The Sr/Ba ratios in all samples vary from 0.20 to 1.72, with a mean of 0.62 (Table 3). The ratios of Sr/Ba are lower than 1.0 except from 710–736 m, which exceeds 1.2. B/Ga values greater than 5.0 indicate salt water, from 3.0 to 5.0 indicate brackish water, and less than 3.0 indicate fresh water^39^. The B/Ga ratios in all samples are 1.99–8.66, with a mean of 4.24 (Table 3). A significant increase of B/Ga values occurs from 710 to 736 m, with values greater than 5.0 (Fig. 7).

Paleoredox conditions. Trace elements such as V, U, Ni, Co, Cr, and Ce are generally considered “redox-sensitive metals” and could be used as paleoredox proxies with minimal detrital influence in lake environments^40^. It has been widely accepted that the trace element ratios of U/Th, Cu/Zn, and V/ (V + Ni) can be used as an index for paleoredox conditions^40^. Hatch and Leventhal documented that an environment characterized by strongly reducing and euxinic conditions led to V/ (V + Ni) ratios greater than 0.84, while that under suboxic-anoxic environments led to V/ (V + Ni) ratios of 0.60–0.84^40,41^. The V/ (V + Ni) ratios for all the Xiahuapidianzi Formation samples vary between 0.64 and 0.79, with a mean of 0.74 (Table 3). A ratio of Cu/Zn greater than 0.63 indicates an oxidizing environment, a value of 0.50–0.63 indicates a weakly oxidizing environment, and a value less than 0.21 indicates a reducing environment. The Cu/Zn ratios for all the Xiahuapidianzi Formation samples vary between 0.20 and 0.52, with a mean of 0.32 (Table 3). Furthermore, U, V and Mo have a variety of chemical valence states, which are significantly affected by the redox state during deposition; most of them are authigenic components in sediments or sedimentary rocks, and almost no migration occurs during diagenesis, keeping the original record of deposition. U and V are reduced and can accumulate under denitrifying conditions, whereas Mo are enriched mainly under sulfate-reducing conditions alone. Based on the difference in geochemical properties of these two elements, U and V enrichment without Mo enrichment, we could infer suboxic/anoxic depositional without free H_2_S. Conversely, sediments exhibiting concurrent enrichments in U, V and Mo reflect euxinic conditions^34^.

Terrigenous detrital. Rivers carry large amounts of terrigenous detrital matter into lakes. Thus, an increase in detrital input probably results from increased river input. The concentrations of high field strength elements, such as Ti (titanium) and Th (thorium), can reflect changes in the input of terrigenous detrital matter. Because Ti (titanium) and Th (thorium) are commonly overwhelmingly of detrital origin and usually immobile during diagenesis^42^. The content of Ti (titanium) and Th (thorium) are 2729–6041 μg/g mean = 4706 μg/g (n = 24) and 5.5–16.7 μg/g (mean = 12.6 μg/g, n = 24) (Table 3).

Palaeoproductivity. The nickel (Ni) and copper (Cu) are dominantly delivered to the sediments in association with OM (organometallic complexes). Moreover, they are often referred to as ‘sulfide forming’, because there are released through OM decay and can be trapped by pyrite if sulfate-reducing conditions prevail. Consequently, the high contents of Ni and Cu in the sediments indicate not only the high organic matter fluid, but also the reducing sedimentary environment. Moreover, even if OM be remineralized by bacterial activity, Ni and Cu could be retained within the sediments, being hosted by pyrite (most frequently). In that way, Ni and Cu may speak to the original presence of OM and consider a representative proxy to OM^34^. The content of Ni and Cu was 26.2–73.0 μg/g, mean = 42.5 μg/g and 29.9–48.5 μg/g, mean = 37.9 μg/g respectively. The barium (Ba) accumulation rate generally shows a positive correlation with the OM content and primary productivity^43^. The content of Ba was 477–1555 μg/g, mean = 704 μg/g (Table 3). The TOC content in the sediment can more sensitively reflect the changes of lake organic carbon production and can be used to estimate the ancient productivity of ancient lakes^44^. The TOC of the Xiahuapidianzi Formation varied from 0.48 to 2.35%, with an average of 1.16%.

## Discussion

### Volcanic activity during OAE1a

The OAE1a is derived by two mechanisms: (1) the release of massive volcanic CO_2_ emission (^13^C ~ − 5 %)^45,46^ and (2) the dissociation of methane hydrates (^13^C ~ − 60 %)^7^. Furthermore, Kuhnt et al. and Yamamoto et al. believe that the negative δ^13^C shift was not short-lived (up to 0.3 Myr) based on the estimated C3 segment duration^47,48^. Moreover, Wang et al. recorded giant Aptian volcanism in the Songliao Basin and Northeast Asia (SB-V) and the Ontong Java plateau (OJP), and it could have contributed to high CO_2_ and climatic change^49^. Other researchers, such as Erba, Larson and Erba, Tarduno et al., identified a prominent negative C-isotope excursion of the OAE1a by analyzing the volcanic rocks within and around the SB-V^5,45,50^. In this study, three volcanic ash layers were found (Fig. 2) within the Xiahuapidianzi Formation (121–118 Ma) as indicated by the intense and frequent volcanic activities during the Xiahuapidianzi Formation deposition. The excursion of pyrite sulfur isotopes (δ^34^S_py_) and the sulfur abundance (TS) in the Xiahuapidianzi Formation (Fig. 3 and Table 1) reflect the exogenous SO_4_^2−^ input, which has been associated with volcanic activities^20^.

### Response of continental lacustrine deposition during OAE1a

**Figure 8:**
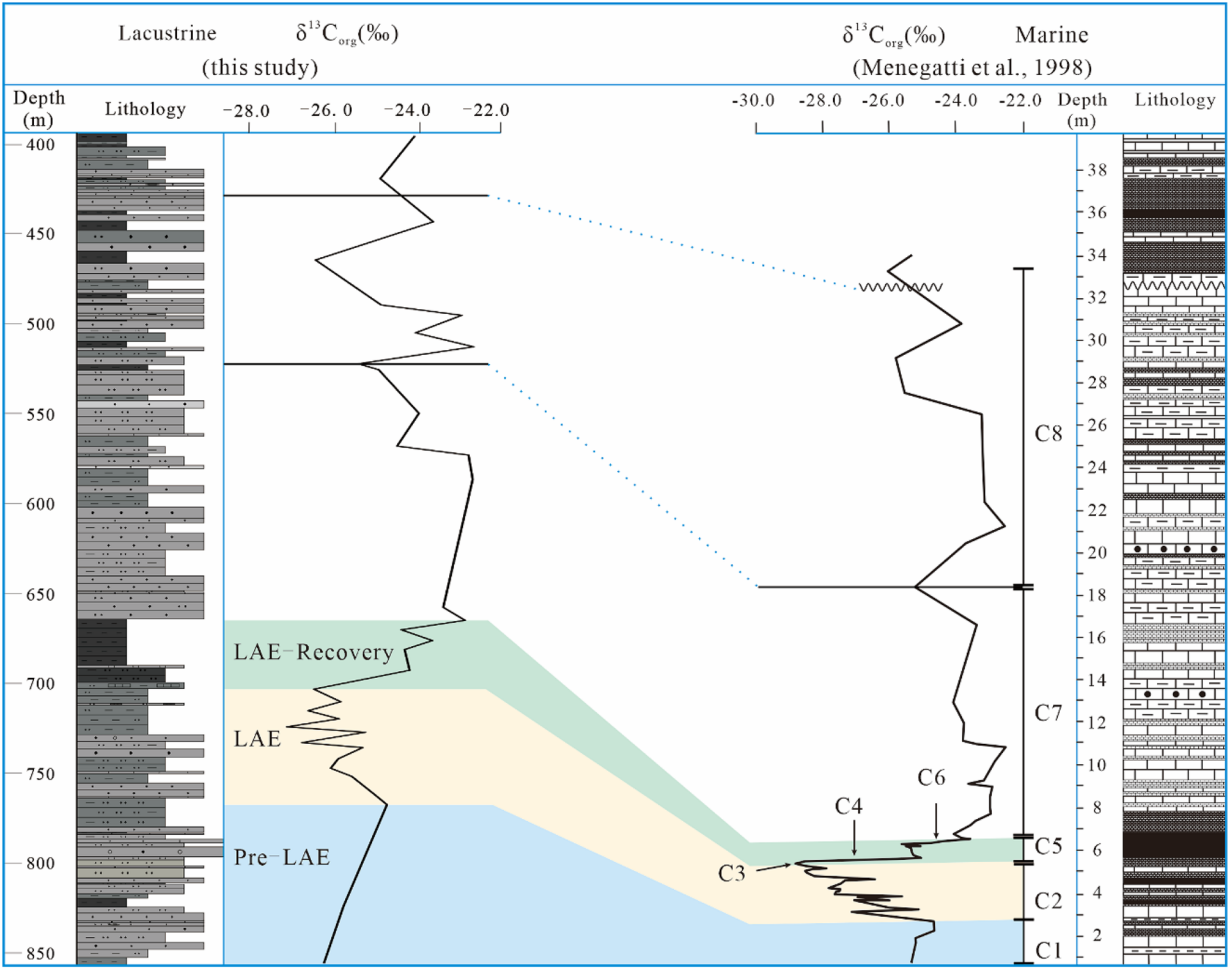
Correlation of the δ^13^C_org_ curve for the continental Xiahuapidainzi Formation (LAE) with marine strata (OAE) from Italy^2^.

Menegatti et al*.* divided the global ocean isotope change trend during OAE1a into C1–C8 stages^2^ (Fig. 8), which have been widely acknowledged^2,47,48,51^. Figure 8 shows that the δ^13^C_org_ curve of the lacustrine Xiahuapidianzi Group can be correlated well with the marine curve (C1–C8) (Fig. 8). We divide the lacustrine anoxic event (LAE) period into the following three phases according to the changes in isotopes (Fig. 3) and trace elements (Fig. 7) to facilitate interpretations of changes in the terrestrial phase under the influence of global anoxia events (LAE) and comparisons with marine changes (OAE) (Fig. 8): Pre-LAE (855.62–767.23 m), LAE (767.23–703.25 m), LAE-recovery (703.25–665.10 m). Detailed explanations are provided as follows.

#### Pre-LAE stage

From depths of 860.00 to 767.23 m (Fig. 8 Pre-LAE), the lithology is argillaceous siltstone, pebbled sandstone, silty mudstone and thin-layer mudstone, thus reflecting a strong hydrodynamic sedimentary environment. The values of δ^13^C_org_ change from − 26.3 to − 24.8 ‰, showing an increase of 1.5‰, which may be related to increased photosynthesis. Studies on modern photosynthetic plants indicated that moderate increases in pCO_2_ are beneficial to bio-photosynthesis^52^. However, if the pCO_2_ gradually increases and exceeds a certain CO_2_ concentration value (e.g., − 20‰ to − 35‰), then photosynthesis will be inhibited or even decease^53^.

Most aquatic organisms use dissolved carbon dioxide as an enzyme substrate. When CO_2_ (aq) is higher, the selectivity of organisms to ^12^C is enhanced, and the δ^13^Corg value is negative excursion. When the high productivity caused by algae in the water body, the consumption of CO_2_ is large and CO_2(aq)_ is low. When CO_2(aq)_ is in short supply, organisms will use ^13^C-rich HCO_3_^-^ (normal lake water temperature HCO_3_^−^ is 7–10‰ heavier than the δ^13^Corg value of dissolved CO_2_) as the enzyme substrate. The δ^13^Corg position excursion may prove this^54^.

The Sr/Cu and Rb/Sr ratios show a warm and humid paleoclimate condition, which is suitable for bio-photosynthesis^55^. Therefore, the increase in carbon isotopes corresponds to increased photosynthesis. The TOC and rare element Ba concentrations are good proxies of paleoproductivity^44^. The TOC varied from 0.43 to 1.43 % (approximately 1.00% increase), and Ba varied from 477.4 to 794.3 µg/g (Table 3), which indicated that the increased photosynthesis led to an increase in paleoproductivity. The Mo abundance (Table 3) further suggested the increasing organic carbon burial in this abnormal interval^56^.

The δ^34^S_py_ value increases from ~11.0 to ~13.4‰ (about 2.4‰) and then decreased to ~6.0. The TS_pyr_ values did not show obvious change (<0.1‰) (Fig. 3 and Table 1). The δ^34^Spy increasing values were mainly caused by OM rising according to the microbial sulfate reduction (MSR) reaction. With OM growth, ^32^SO_4_^2−^ will be consumed, which in turn leads to increases in δ^34^S_py_, show a reducing environment. However, the decrease of δ^34^S_py_ (after the short increase) may suggest an external SO_4_^2−^ input, which Berner ascribed to volcanic activities^20^.

The average Pr/Ph value is 0.59 at 860.00 m and 0.25 at 798.00 m, thus indicating a reducing environment, which was probably caused by the promotion of pCO_2_. The values of V/(V + Ni) < 0.84 and Cu/Zn < 0.52 also indicated a suboxic-dysoxic environment (Table 3). The distribution patterns of n-alkanes are considered good indicators of the depositional environment and OM source. Peters et al. demonstrated that n-alkane ≤ nC19 is primarily associated with planktonic organisms and/or bacterial communities^18^. Cranwell thought that nC20-nC25 are mainly derived from aquatic macrophytes both marine and non-marine^57^ and > nC25 are mainly derived from terrestrial plants^18,58,59^. The n-alkane distribution (860.00 m and 798.00 m in Fig. 5) of the mudstone are < nC25, indicating mixed OM source inputs from planktonic organisms, bacterial communities and aquatic macrophytes at depths from 860.00 m to 767.23 m.

The biological pump^65^ can well explain the CO_2_ conversion to OM by the process [CO_2_ + H_2_O − CH_2_O (POC) + O_2_]^66^. Large amounts of CO_2_ were converted to particulate organic carbon (POC) via the biological pump. As CO_2_ was consumed, the OM increased in this process. With more organic matter produced in the surface water, it consumes lots of oxygen to degradation before the settlement which will aggravate the formation of suboxic-dysoxic environment.

The initial volcanic activity moderately enhanced pCO_2_ and important nutrient fluxes (such as biolimiting metals) to the continental lake, which promoted plant photosynthesis. which sustained high biological productivity^3,45^. The productivity increased in the short-term, and the TOC gradually increased. At this stage, the environment was mainly warm and humid sedimentary, and the water bodies were mainly low-oxygen freshwater. Based on these results and analyses, a pattern diagram of the pre-LAE stage (Fig. 8. pre-LAE) was established.

#### LAE stage

The LAE stage represents the zone at depths between 767.38 and 703.25 m (Fig. 8 LAE). The lithology is mainly silty mudstone, calcareous mudstone and argillaceous siltstone interbedded, and the hydrodynamic environment is lower than that of the previous stage. The TIC content of calcareous mudstone increased significantly and was within the range of 3.38–48.0%. Bischoff and Cummins documented that calcareous mudstone mainly formed in low-lying areas, which experienced seasonal rains and intense evaporation caused by an arid or semiarid climate^60^. In arid climate conditions, the atmospheric precipitation decreases, the water quality becomes salty, the pH value is higher, the chemical precipitation of calcite is enhanced, and "calcium" is formed. The solubility of calcium carbonate increases with the depth of water. At a certain depth of water, the rate of dissolution of carbonate will be equal to the rate of precipitation. This depth is called carbonate compensation depth (CCD). When the lake bottom is above the compensation depth of carbonate, calcium carbonate is easy to precipitate to form a calcareous layer. Otherwise, the calcium carbonate will dissolve and the calcareous layer cannot be formed. Therefore, the content of calcareous components can reflect changes in lake level up and down or changes in water depth, evolving from a pure mudstone layer to a calcareous layer, and the water depth may have a shallower trend. These may all reflect the change from warm to hot climate.

The δ^13^C_org_ values range from − 24.8 to − 27.2‰, indicating an approximately 2.4‰ maximum decrease (Fig. 3 and Table 1). The negative excursion of carbon isotope is a typical case of OAE1a^2,46,61^. The previous studies on marine isotopes (Fig. 8) indicated an isotopic decline of ~ 3 ‰ in the C2-C3 stage^2^. There is little doubt that the δ^13^C_org_ variations in this stage may have resulted from the early Aptian global carbon disturbance. The volcanic activity during this period is probably the main reason for the ~ 2.4‰ carbon isotopic decline in the study area. This assumption is supported by the high atmospheric pCO_2_ content during the Cretaceous due to the Ontong Java and SB-V volcanic activities^6,49,50,56^, which may contribute to the global greenhouse^45,46,50,61^.

As the proxy of paleoclimate, the ratios of Sr/Cu and Rb/Sr (Table 3 and Fig. 7) rise up, which reflect a semi-humid to hot arid climate. But the ratios of Sr/Cu and Rb/Sr could be affected by the ion substitution due to similar radius of Sr and Ca^62^. Therefore, we combine biomarker and the enrichment factor (EF) for comprehensive analysis and verification the paleoclimate variation.

The n-alkane is C19 at 772.7 m and C17 at 720 m (Fig. 5), which are characterized predominantly by planktonic organisms and/or bacterial communities and little with terrestrial high plants. The C29 (m/z = 217, depth = 720.00) in Fig. 5 show an obvious low, reflect the litter source of terrestrial plants input. The biomarker results reflect that the decrease of terrestrial input, which may cause by the change of climate from warm and humid to hot and arid.

**Figure 9:**
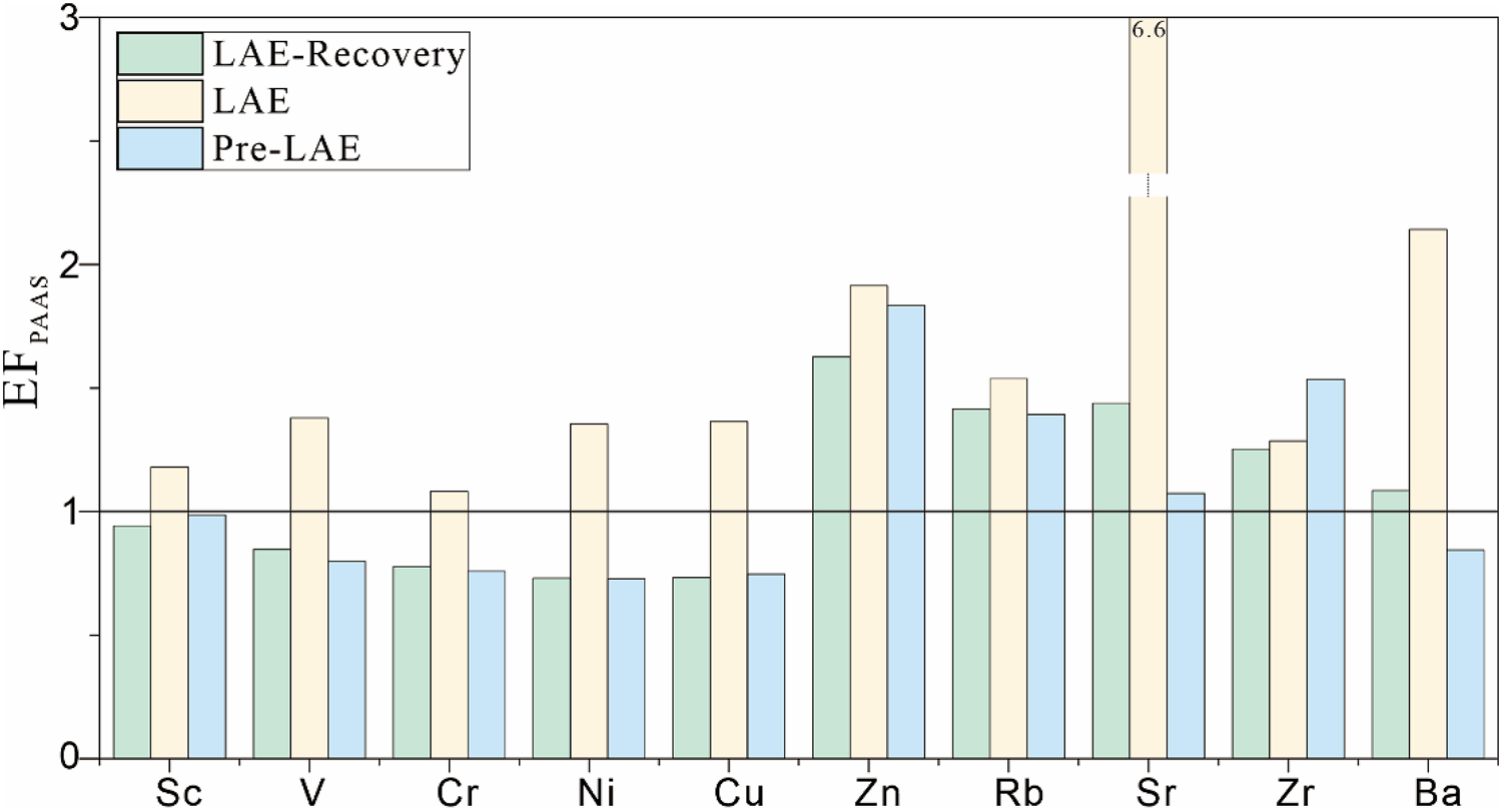
Enrichment factors (EF) results according to post Archean average Australian shale (PASS) standard.

The enrichment factor (EF) could well eliminate the dilute effect of the biogenic carbonate rocks. The enrichment factor of a certain trace element can be expressed as: element factor (EF) = (C/X)_sample_/(C/X)_PAAS_, C = element, X = usually are Al or Th (PAAS = post Archean average Australian shale^63^). If the enrichment factor is greater than 1, it indicates that the element is enriched, otherwise it is depleted. Th only presents four valences, generally not easily soluble in water, and inactive under oxidative conditions, so Th is inactive or very low in the epigenetic zone. The Sr_EF,_ Cu_EF_ and Ba_EF_ increase in LAE stage (Fig. 9), show a hot and arid climate change. The ratio of B/Ga was originally reported to be related to water salinity^30,33,41^, and the average ratios were 6.69, which reflect high evaporation and increased salinity in the shrinking water body. The terrigenous detrital proxy, Zr, Ti, Th (Table 3 and Fig. 7) show a significant decrease at this stage. The increase in salinity and reduce of terrestrial debris also verify the hot and arid climate changes in the depositional environment together with calcareous mudstone petrographic analysis. Zhang, X., and Li, S got the same conclusions (hot and arid climate condition) on mudstone and shale samples by the methods of trace elements, quantitative palaeosalinity, carbon and oxygen isotopes in Jiaolai Basin, Shandong Province, Easten China^37^.

The values of δ^34^S_py_ range from 6.1 to 16.4‰, which indicates an approximately 10.3 ‰ increase. The TS_pyr_ values are between 0.20 and 1.35 ‰ (approximately 1.15 ‰ increase, Table 1), the high TS-pyr content and the increased δ^34^S_py_ reflected isotope fractionation caused by the mineralization of OM (MSR). With increasing δ^34^SO_4_^2−^, the δ^34^SO_4_^2−^ values for pyrite will gradually increase and even exceed the sulfate level of the lake water. This MSR reaction further reflected a reducing environment^64^. The high TS content may imply euxinic environment, support by the enrichment of U, V and Mo (Fig. 7), which could infer euxinic deposition environment with H_2_S.

The TOC values increase by 1.04% (from 0.71 to 1.75%). The content of, Ni, Cu increasing, indicate a high paleoproductivity which mainly contribute from planktonic organisms and/or bacterial communities differ from Pre-LAE stage. The Pr/Ph ratio value was 1.01 at 720.0 m, the Cu/Zn ratio was 0.50–0.63, which further indicated an anoxic condition.

**Figure 10:**
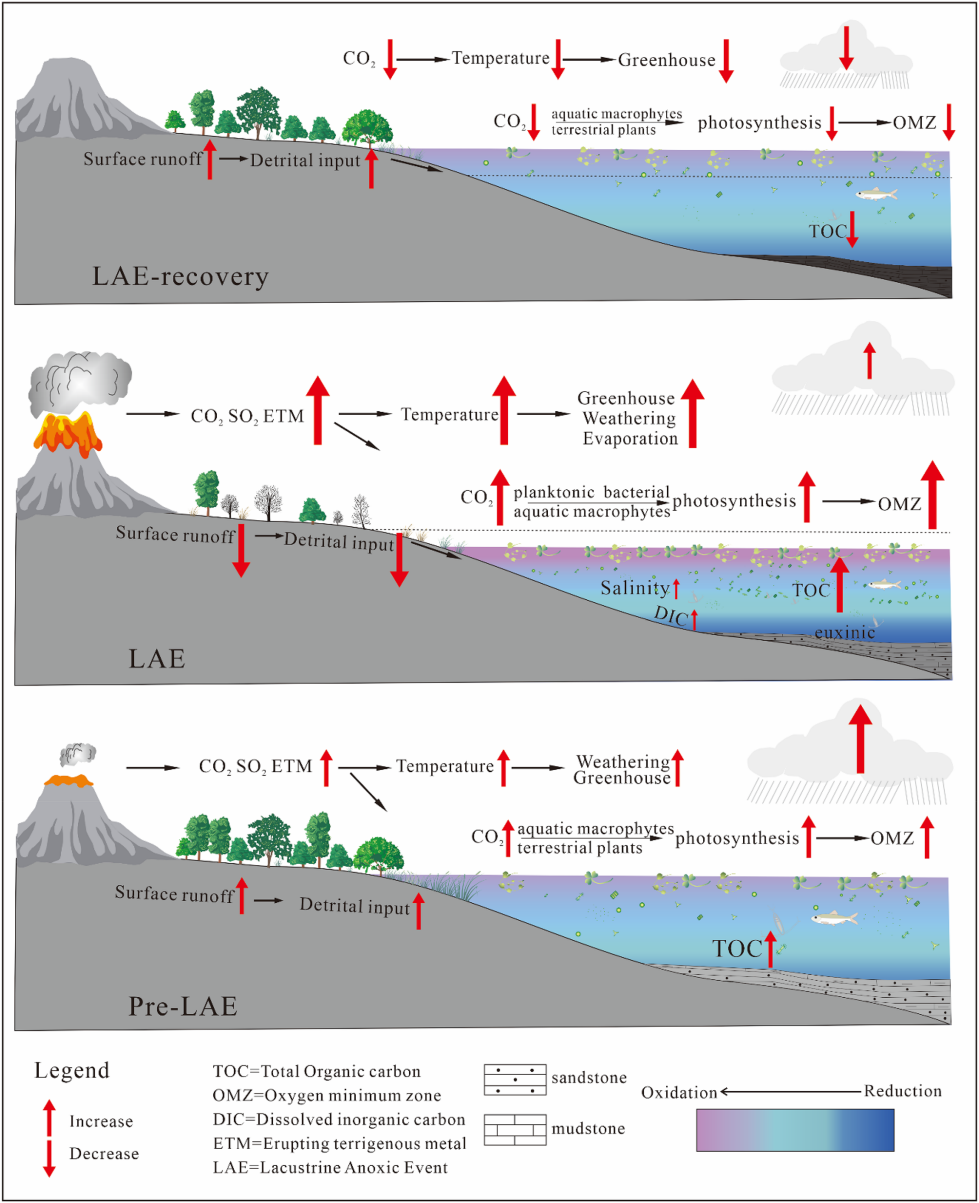
Change model of the different sediment stages (Pre-LAE, LAE, and LAE-recovery) of the continental lake under the influence of the OAE1a period (Drawn by Daijun Fan and Yibo Wang).

The warm humid to hot arid climate weather due to the high release of pCO_2_ by intense volcanic activity. The euxinic deposition environment provide a favorable environment for the storage of organic matter. Based on the above evidence and analyses, a pattern diagram of the LAE stage (Fig. 10 LAE) was established.

#### LAE-recovery stage

The third stage at 703.25 m and 665.10 m (Fig. 8 LAE-recovery) mainly included black mud deposits in a lacustrine sedimentary environment. In this stage, the δ^13^C_org_ of the Xiahuapidianzi Group has undergone a successive two-stage increase with the marine C4-C6 stages (Fig. 8) after the intense decrease of δ^13^C_org_. The marine C4-C6 stages usually accompany black mudstone deposits (Livello Selli). A 40 m thick black mudstone deposit was observed in the OAE-recovery stage (Fig. 8), which shows a deeper and more stable sedimentary environment than the OAE stage. The value of δ^13^C_org_ is within the range of -26.5 ‰ to -22.9 ‰, which indicates an increase of 2.6 ‰. The increase in the δ^13^C_org_ isotope reflected the continuous consumption of CO_2_ to produce OM and the termination of OAE 1a under the less active (or ceased) SB-V and OJP volcanism^49^.

The value of δ^34^S_py_ is in the range of 3.7 to 9.1 ‰, and the value of TS_pyr_ is between 0.42 ‰ and 0.93 ‰ (Table 1). δ^34^S_py_ and TS_pyr_ still show increases as observed in the LAE stage, although the change is less than that in the LAE stage (Fig. 3) because less OM is available to support the MSR reaction. The decreased TOC value and Ba, Ni, Cu content indicate a decrease in paleoproductivity as the pCO_2_ value declined. A large amount of CO_2_ consumption also led to paleoclimate changes from an arid hot to warm humid climate according to the decreased ratios of Sr/Cu and Rb/Sr (Fig. 7). In the warm humid climate, the detrital input increased and paleosalinity of the lake decreased as indicated by changes in the Zr, Ti, and Th contents and Sr/Ba and B/Ga ratios (Table 3). The ratios of Cu/Zn, U/Th, and V/V(V + Ni) indicate suboxic-anoxic paleoredox conditions.

The n-alkanes distribution (C17 to C23) (Fig. 5) reflected the change in OM source from mixed resources^18,58,59^. Based on these evidences and analyses, a pattern diagram of the LAE-recovery stage (Fig. 10 LAE recovery) was established.

## Conclusions

We report a new lacustrine anoxic event (LAE) under the influence of the global OAE1a event. The carbon isotope stratigraphic comparison and chronological data show that the Xiahuapidianzi Formation in the Hongmiaozi Basin is a valuable terrestrial research region for studying extreme climate changes (e.g., greenhouse and anoxic) in the Cretaceous.

The comprehensive analysis (e.g., stable carbon sulfur isotope, GC–MS and rare element analyses) of the Xiahuapidianzi Formation indicated that during the deposition period, volcanic activity led to the input of a large amount of greenhouse gases (CO_2_), sulfate, nutrients, etc., which resulted in a significant decrease of carbon and sulfur isotopes. Due to the large amount of greenhouse gases (high pCO_2_), the climate was significantly warm, even dry and hot climate conditions. Under these conditions, the surface runoff decreased, the lake evaporation and salinity increased, calcareous mudstone was deposited. Moreover, the dry and hot climate and high salinity may lead to organisms to die and quickly accumulate to form an anoxic to euxinic environment, which are beneficial to the preservation of OM (2.0% TOC). In the LAE-recovery stage, lake expansion, high productivity and rapid OM accumulation led to the favorable suboxic-anoxic environment for thick black mudstone storage.
